# Identification of polymorphic SVA retrotransposons using a mobile element scanning method for SVA (ME-Scan-SVA)

**DOI:** 10.1186/s13100-016-0072-x

**Published:** 2016-07-30

**Authors:** Hongseok Ha, Jui Wan Loh, Jinchuan Xing

**Affiliations:** 1 Department of Genetics, The State University of New Jersey, Piscataway, 08854 NJ USA; 2 Human Genetic Institute of New Jersey, Rutgers, The State University of New Jersey, Piscataway, 08854 NJ USA

**Keywords:** SVA, Retrotransposon, High-throughput sequencing, ME-Scan

## Abstract

**Background:**

Mobile element insertions are a major source of human genomic variation. SVA (SINE-R/VNTR/Alu) is the youngest retrotransposon family in the human genome and a number of diseases are known to be caused by SVA insertions. However, inter-individual genomic variations generated by SVA insertions and their impacts have not been studied extensively due to the difficulty in identifying polymorphic SVA insertions.

**Results:**

To systematically identify SVA insertions at the population level and assess their genomic impact, we developed a mobile element scanning (ME-Scan) protocol we called ME-Scan-SVA. Using a nested SVA-specific PCR enrichment method, ME-Scan-SVA selectively amplify the 5′ end of SVA elements and their flanking genomic regions. To demonstrate the utility of the protocol, we constructed and sequenced a ME-Scan-SVA library of 21 individuals and analyzed the data using a new analysis pipeline designed for the protocol. Overall, the method achieved high SVA-specificity and over >90 % of the sequenced reads are from SVA insertions. The method also had high sensitivity (>90 %) for fixed SVA insertions that contain the SVA-specific primer-binding sites in the reference genome. Using candidate locus selection criteria that are expected to have a 90 % sensitivity, we identified 151 and 29 novel polymorphic SVA candidates under relaxed and stringent cutoffs, respectively (average 12 and 2 per individual). For six polymorphic SVAs that we were able to validate by PCR, the average individual genotype accuracy is 92 %, demonstrating a high accuracy of the computational genotype calling pipeline.

**Conclusions:**

The new approach allows identifying novel SVA insertions using high-throughput sequencing. It is cost-effective and can be applied in large-scale population study. It also can be applied for detecting potential active SVA elements, and somatic SVA retrotransposition events in different tissues or developmental stages.

**Electronic supplementary material:**

The online version of this article (doi:10.1186/s13100-016-0072-x) contains supplementary material, which is available to authorized users.

## Background

Mobile elements are discrete DNA fragments that can move and integrate into other locations in a genome. More than two-thirds of human genome are occupied by repetitive or repeat-derived sequences, including active mobile elements that are still capable of transposition [1]. Mobile elements can insert and disrupt host genes or participate in genomic rearrangement, resulting in diseases (for review, see [2–4]). In humans, some mobile element insertions (MEIs) are polymorphic across individuals [4, 5]. Besides their functional and structural genomic impact, these polymorphic MEIs (pMEIs) are also important markers for ascertaining human population relationships and evolutionary history [6–8]. Therefore, it is of great interest to identify pMEIs in human populations. In general, there are two high-throughput sequencing based strategies for identifying pMEIs; whole genome and MEI-targeted sequencing. Compared with whole genome sequencing, MEI-targeted high-throughput sequencing methods are more cost-effective [9]. Although a number of targeted high-throughput sequencing methods have been developed for *Alu* and L1 elements [10–14], to date the only targeted sequencing method for SVA (SINE-R/VNTR/Alu) elements is retrotransposon capture sequencing (RC-seq) [10, 15–17].

**Fig. 1:**
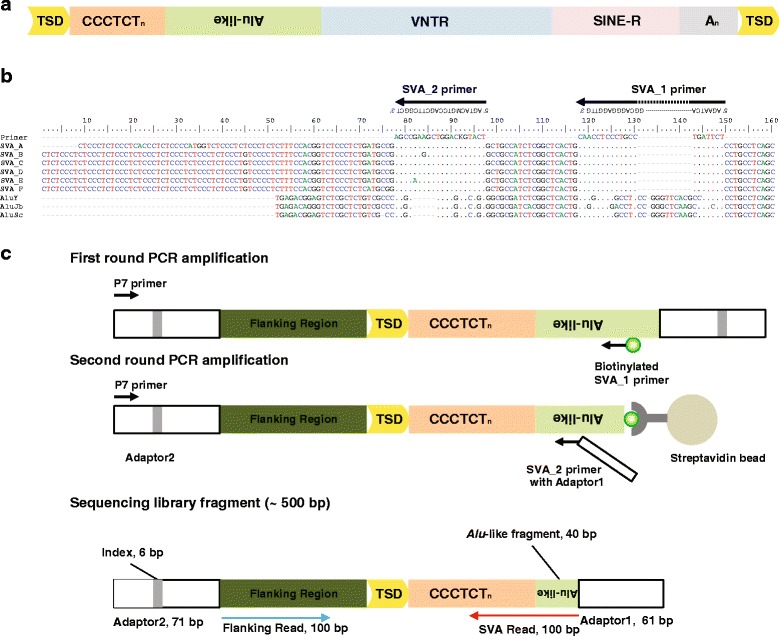
Experimental protocol design. **a** Scheme of SVA element structure. **b** Sequence alignment of SVA_1 and SVA_2 primer binding sites and SVA, *Alu* subfamily consensuses. The SVA_1 and SVA_2 primer sequences are shown above of the alignment and the amplification directions are indicated by arrows. Top row of the sequence alignment shows the sequences of the primer binding sites of SVA_1 and SVA_2. SVA_1 binding site includes the SVA characteristic deletion as compared to *Alu* sequences. Dots in the alignment represent the same nucleotides as the primer binding site sequences. Deletions are shown as dashes and mutations are shown as the correct base for the consensus. **c** SVA-specific amplifications during ME-Scan-SVA library construction and the final DNA fragment structure. The DNA library after second-round amplification is size-selected at ~500 bp (an example electropherogram image is shown). White box: adaptor; grey box: index; dark green box: flanking genomic region; yellow box: TSD; orange box: (CCCTCT)_n_ hexamer simple repeat; light green box: SVA *Alu*-like region

SVA is a composite element consisting of a (CCCTCT)_n_ hexamer simple repeat region at the 5′ end, an *Alu*-like region, a variable number of tandem repeats (VNTR) region, a short interspersed element of retroviral origin (SINE-R) region, and a poly-A tail after the putative polyadenylation signal (Fig. 1a). SVA insertions have all the hallmarks of L1-mediated target primed reverse transcription, such as poly(A) tail, target-site duplications (TSDs), 5′ truncation, and have been shown to mobilize by hijacking the L1-encoded protein machinery [18–21]. SVA elements represent the youngest retrotransposon family in the human genome and many insertions are polymorphic among human populations [5, 18, 22]. The polymorphism rates of members of the youngest subfamilies SVA_E and SVA_F were estimated as 37.5 and 27.6 %, respectively [18].

Although SVA elements only constitute approximately 0.1 % of the human genome, they have substantial biological impact in human. Insertion of SVA elements can trigger exonization, polyadenylation, enhancer and alternative promoter events, which lead to the formation of various transcript isoforms and evolutionary dynamics that contributes to the differences in gene expression level [19, 23–28]. Several human diseases have been attributed to SVA insertions or SVA-associated deletions, including Fukuyama congenital muscular dystrophy, Lynch syndrome, X-linked agammaglobulinemia, autosomal recessive hypercholesterolemia, hemophilia B, and neurofibromatosis type 1 [29–33]. Therefore, it is important to systematically analyze polymorphic SVA insertions in human populations.

Mobile element scanning (ME-Scan) is a targeted high-throughput sequencing strategy for MEIs. In previous studies, the technique was applied for identifying AluYb8/9 insertion polymorphisms in human genomes [11, 14], and Ves SINE insertions in bat genomes [34]. In this study, we developed a ME-Scan method and an associated data analysis pipeline for SVA elements, which we termed ME-Scan-SVA. We then demonstrated the method by examining SVA insertions in 21 individuals.

## Results

### ME-Scan-SVA overview

#### Experimental protocol design

We designed a two-round nested PCR amplification protocol for SVA following the existing ME-Scan method [35]. We targeted the 5′ *Alu*-like region of the SVA elements to selectively enrich for SVA elements. Despite the high similarity between the SVA *Alu*-like region and *Alu* subfamily consensus sequences, one insertion and one deletion are shared by all SVA sequences (Fig. 1b). Therefore we designed SVA-specific primers in these regions. A biotinylated primer (SVA_1) was used for the first round PCR reaction and the second-round nested primer (SVA_2) was used to further improve specificity and add Illumina sequencing adaptors. Because typical SVA truncations happen at the 5′ of the insertion, this nested-PCR design at the 5′ end of the SVA element allows us to selectively enrich full-length SVA elements. In addition, 5′ or 3′ truncated SVA elements that contains both SVA_1 and SVA_2 primer binding sites (Fig. 1b, SVA consensus position 78 - 137) will also be amplified. Based on the human reference genome (hg19), we estimate that this method can amplify 65 % of SVA_D (828/1274), 27 % of SVA_E (52/192), and 24 % of SVA_F elements (198/821), respectively.

A DNA fragment in the final sequencing library contains a variable-length 5′ flanking genomic sequence, the 5′ terminus of an SVA element ends at the primer binding site of SVA_2, and 132 base pair (bp) of sequencing adapters that flank either end of the fragment (Fig. 1c bottom). The expected SVA fragment size is the size of the (CCCTCT)_n_ hexamer simple repeats plus 40 bp in the *Alu*-like region. Because of the variable size of the simple repeat and possible the 5′ truncation, the size of an SVA fragment could vary between 20 bp (SVA_2 primer binding site only) to several hundred bps. We aim to minimize the library size for sequencing efficiency while maintaining sufficient flanking sequence for identifying the genomic location of the SVA insertions. Therefore, we first fragment the genomic DNA to about 1,000 bp in size. After library construction, we select DNA fragments around 500 bp for sequencing (~130 bp adaptor sequence + ~370 bp SVA sequences and genomic flanking sequence).

#### Computational analysis pipeline

**Fig. 2:**
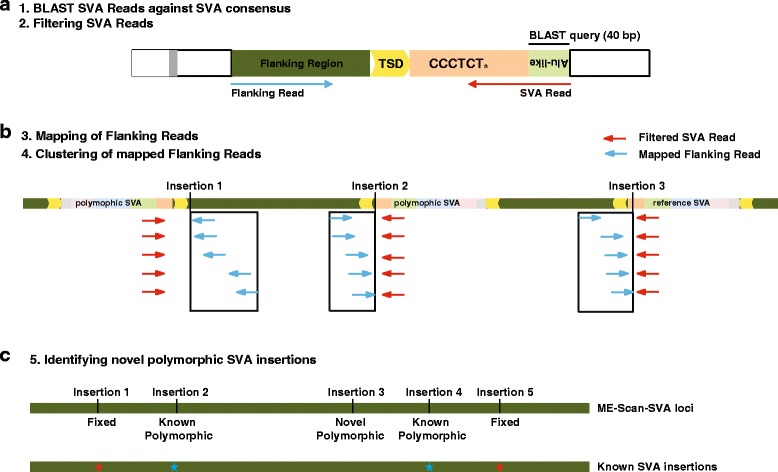
Computational data analyses pipeline. **a** BLAST-based SVA Read filtering. Location of the 40 bp BLAST query sequence in the *Alu*-like region in the SVA Read is labelled. The two pair-end sequencing reads are represented by red arrow (SVA Read) and blue arrow (Flanking Read), respectively. **b** Flanking Read mapping and clustering. After mapping by BWA-MEM, Flanking Reads are filtered based on mapping quality score. Filtered Flanking reads that are within a 500 bp sliding window are then clustered into candidate insertion positions. The color scheme is same as Fig. 1. Black box: clustering window. **c** Identifying different types of SVA insertions. A representative genomic region (dark green box) is shown. Top row: SVA insertions identified by ME-Scan-SVA. Bottom row: known SVA annotated in the reference genome. Red star: fixed SVA insertions in the reference genome; blue star: known polymorphic SVA insertions

**Fig. 3:**
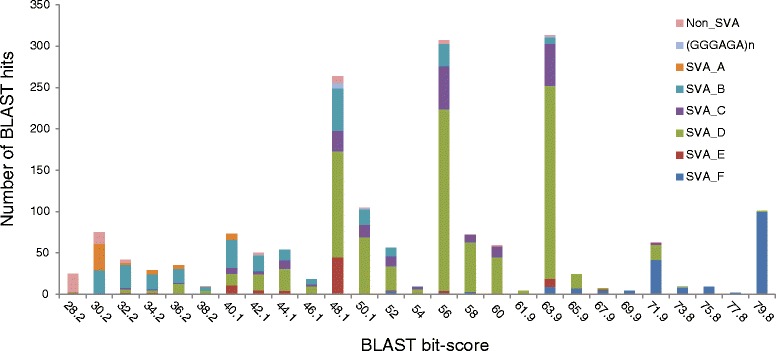
Distribution of BLAST bit-scores of the 40 bp *Alu*-like fragments in SVAs in the human reference genome. X-axis: BLAST bit-scores, Y-axis: the number of SVAs in the human reference genome (hg19) in each bit-score category. Each bar is broken down into color sections based on the RepeatMasker annotation

We designed a pipeline for ME-Scan-SVA analysis based on the general ME-Scan workflow [35]. Figure 2 shows an outline for the analysis pipeline. Using the Illumina 100 bp pair-end sequencing format, two sequencing reads are generated from each DNA fragment (Fig. 2a). We use the 40 bp *Alu*-like region in the first read (referred as the SVA Read in the following text) to determine if a read-pair is derived from an SVA locus (Fig. 2a). For each SVA Read, the *Alu*-like region is compared with the SVA consensus sequence [36] using BLAST [37] and the resulted bit-scores are recorded. The BLAST bit-score is a normalized measurement of the similarity between the SVA Read and the corresponding SVA consensus sequence. To choose a suitable cutoff for the BLAST bit-score, we determined the BLAST score distribution of SVA sequences in the human reference genome (Fig. 3). As expected, almost all SVAs from SVA_F, the youngest SVA subfamily, are present in the highest BLAST bit-score bins (>65). The majority of SVAs in the subfamilies SVA_D, SVA_E, and SVA_F have BLAST bit-scores higher than 48. Because these three subfamilies contain all known polymorphic SVA insertions, we selected BLAST bit-score 48 as a relaxed cutoff and 65 as a stringent cutoff. The relaxed cutoff is expected to capture more candidate loci. The stringent cutoff will enrich for the youngest subfamily SVA_F, which is expected to contain higher proportion of very recent insertions (Fig. 3). We then filter SVA Read based on selected bit-score cutoffs. A typical 100 bp SVA Read contains 40 bp SVA *Alu*-like region, and the variable (CCCTCT)_n_ hexamer simple repeats region. Because the simple repeat region are often longer than 50 bp in size, most of the SVA Reads are expected to contain little or no flanking genomic sequences. Therefore, we use the second read in the read pair (referred as the Flanking Read in the following text) to identify the genomic location of an SVA insertion. Flanking Read sequences are aligned to the reference genome using the program BWA-MEM (Burrows-Wheeler Alignment Tool- maximal exact matches) [38]. The mapped Flanking Reads are then filtered based on their mapping quality scores to ensure the high-confidence mapping of the read. After mapping, the end positions of the mapped Flanking Reads are sorted, and then clustered within a sliding window of 500 bp in size. Within each cluster, the Flanking Read mapping position that is closest to the SVA insertion site is chosen as the insertion position for that locus (Fig. 2b). Depending on the length of the SVA element in the DNA fragment, the Flanking Reads might not cover the exact SVA insertion site. The candidate SVA insertion loci are then separated into several types (Fig. 2c). Reference SVAs are loci that are annotated by RepeatMasker in the human reference genome and passed the BLAST score cutoff. Fixed SVAs are reference SVA loci that are not known to be polymorphic. Known polymorphic SVAs are loci reported in previous studies [5, 22, 39, 40]. Finally, novel polymorphic SVA insertions are loci that do not overlap reference and known polymorphic SVAs.

### Applying ME-Scan-SVA to 21 human samples

#### Data generation

To demonstrate the feasibility of our protocol, we constructed a ME-Scan-SVA library using 21 individuals from two HapMap populations, including six parent-offspring trios (Table 1). All samples were pooled after indexing and the pooled library was used to construct a ME-Scan-SVA sequencing library. The library was sequenced using the Illumina Hiseq 2000 with 100 bp paired-end format. We obtained 152.9 million total read pairs from the library, and the average and median of individual read number is 7.3 and 6.3 million, respectively (Additional file 1: Table S1).

**Table 1:** The cutoffs used and the number of SVA loci identified in each individual

				Relaxed				Stringent			
Individual	Population	Family	Relation	Cutoff (TPM,UR)	All	Poly-morphic	Novel	Cutoff (TPM,UR)	All	Poly-morphic	Novel
NA12872	CEPH	1459	paternal grandfather	(5,10)	1388	157	15	(16,10)	254	68	1
NA12873	CEPH	1459	paternal grandmother	(5,10)	1383	159	6	(14,10)	252	64	1
NA12864	CEPH	1459	father	(5,10)	1407	178	15	(12,10)	263	76	2
NA12874	CEPH	1459	maternal grandfather	(3,4)	1394	169	16	(4,6)	339	156	0
NA12875	CEPH	1459	maternal grandmother	(4,10)	1407	174	12	(13,10)	263	73	4
NA12865	CEPH	1459	mother	(4,10)	1399	171	13	(9,10)	270	80	1
NA12891	CEPH	1463	maternal grandfather	(4,10)	1394	164	10	(11,10)	266	76	1
NA12892	CEPH	1463	maternal grandmother	(5,10)	1387	158	6	(11,10)	265	75	0
NA12878	CEPH	1463	mother	(4,10)	1397	167	13	(13,10)	262	73	1
NA18501	YRI	Y004	father	(3,10)	1399	178	12	(4,10)	390	202	4
NA18502	YRI	Y004	mother	(5,10)	1398	173	14	(11,10)	271	83	2
NA18500	YRI	Y004	child	(4,9)	1401	167	13	(9,10)	285	96	4
NA18504	YRI	Y005	father	(3,10)	1398	168	7	(9,10)	283	92	3
NA18505	YRI	Y005	mother	(4,10)	1408	175	9	(15,10)	268	80	2
NA18503	YRI	Y005	child	(4,10)	1393	167	10	(11,10)	277	87	3
NA18507	YRI	Y009	father	(3,10)	1408	176	11	(11,10)	268	80	3
NA18508	YRI	Y009	mother	(5,10)	1408	175	15	(10,10)	276	87	2
NA18506	YRI	Y009	child	(3,7)	1404	177	13	(10,10)	270	84	2
NA18517	YRI	Y013	mother	(4,10)	1420	185	23	(10,10)	268	82	5
NA18515	YRI	Y013	child	(5,10)	1394	162	13	(17,10)	261	73	2
NA18521	YRI	Y016	child	(6,10)	1388	161	14	(9,10)	280	91	3
Total					1722	428	151		521	310	29

#### Read filtering and candidate loci identification

As described in the “Computational pipeline” section, we filtered SVA Read based on BLAST bit-score cutoffs. We used BLAST bit-score 48 as a relaxed cutoff and 65 as a stringent cutoff. Using the relaxed and stringent cutoffs, 93.8 and 17.6 % of the SVA Read passed the cutoff, respectively (Additional file 1: Table S1).

The vast majority (99.2 %) of Flanking Reads was mapped to the reference genome. More than 82 % of the reads in each individual passed a BWA-MEM mapping quality score cutoff of 29. We used this mapping quality cutoff to exclude low-quality reads and reads that mapped to multiple genomic locations. Overall, 78.1 and 14.5 % of the read-pairs passed both SVA Read and Flanking Read filtering under the relaxed and stringent SVA Read cutoffs, respectively (Additional file 1: Table S1).

To obtain candidate SVA insertion loci, the mapping positions of mapped Flanking Reads were sorted and then clustered within a sliding window of 500 bp in size (Fig. 2b). A total of 28,130 and 7,972 insertion positions were generated from the 21 individuals under relaxed and stringent SVA Read cutoffs, respectively.

#### Sensitivity analysis

To estimate the sensitivity of ME-Scan-SVA, we first identified presumed fixed SVA insertion loci in the human reference genome. The presumed fixed SVA insertion loci are defined as SVA insertions that are present in the reference genome and are known to be not polymorphic in previous studies [5, 22, 39, 40]. Using the relaxed and stringent SVA Read cutoffs, we identified 1,343 and 200 loci as presumed fixed SVAs, respectively. Using this set of SVA insertion loci, we calculated the depth of coverage and the number of unique reads (URs) for each locus. To account for inter-library variation, we normalized the depth of coverage at each locus by the total number of mapped reads in each individual as TPM (tags per million).

**Fig. 4:**
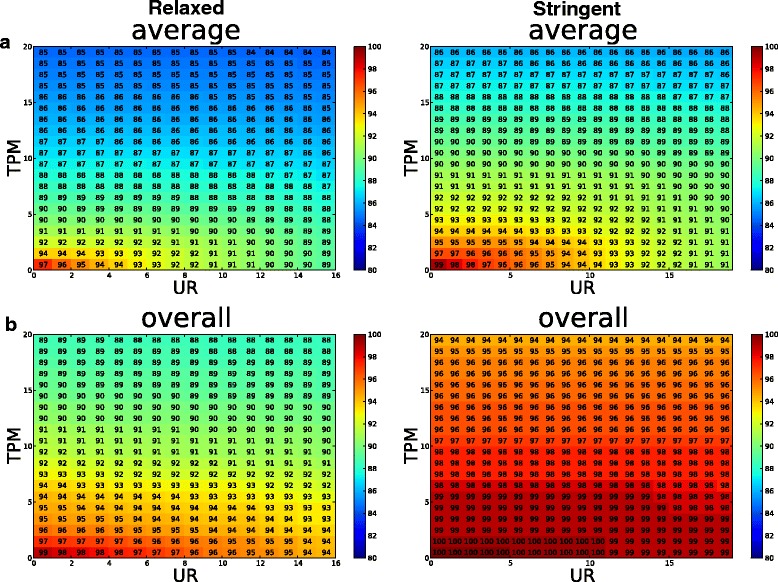
Sensitivity analysis. The sensitivity for identifying fixed SVA insertions under different TPM and UR cutoffs. **a** average individual sensitivity; **b** overall sensitivity. The sensitivity is shown as the percentage of fixed insertions identified. Results under relaxed and stringent SVA Read cutoffs are shown in the left and right panel, respectively

Using the TPM and UR info for each locus, we calculated the sensitivity for identifying fixed loci under different TPM and UR cutoffs (Fig. 4). Overall, we achieve high sensitivity: even at a stringent TPM/UR cutoff 15/15, the pooled data has 89 and 96 % sensitivity, for the relaxed and stringent conditions, respectively (Fig. 4). Among individuals, the sensitivities are similar but lower than pooled data at high cutoffs (Additional file 2: Figure S1).

#### SVA candidate loci identification and validation

**Fig. 5:**
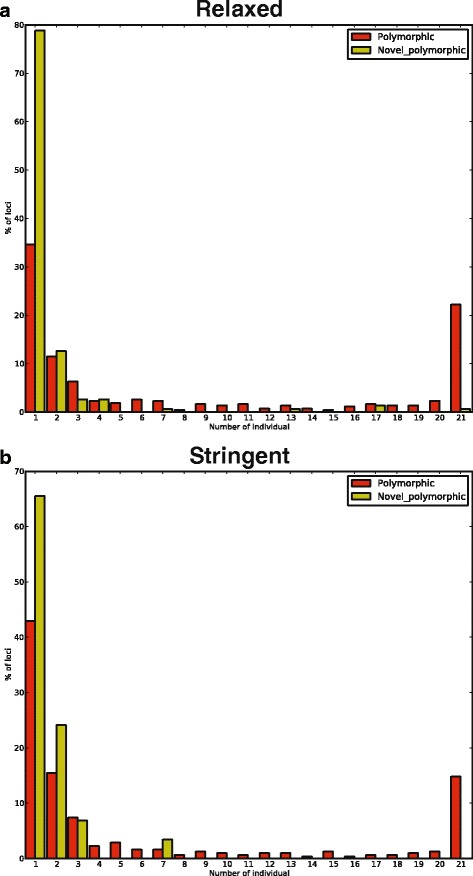
Allele frequency distribution of polymorphic SVA insertions. The number of individuals having an SVA insertion is shown on the X-axis. The percentage of polymorphic or novel polymorphic SVAs in each individual bin is shown on the Y-axis. **a** relaxed SVA Read cutoff; **b** stringent SVA Read cutoff

To identify SVA insertion candidates, we started from the list of candidate insertion positions and used TPM/UR cutoffs that achieve 90 % sensitivity in each individual based on the presumably fixed SVA insertions (Table 1). In each individual, ~1,400/~300 SVA insertion loci were selected under the relaxed/stringent conditions. Among them, ~200/~100 loci are polymorphic, and ~10/2 loci are novel (Table 1). In total, 428 polymorphic SVAs were identified among the 21 individuals under relaxed condition, and 151 of them are novel. As expected, the vast majority of novel insertions are rare, and ~80 % of the loci are only present in one sample. In comparison, some of the known polymorphic loci are more common and are present in all individuals in our dataset (Fig. 5a). Candidate loci from the stringent cutoff exhibit similar allele frequency pattern (Fig. 5b). The final relaxed and stringent call sets are available in Additional files 3 and 4.

To validate polymorphic SVA insertions, we performed PCR validation on 11 candidates (Additional files 5 and 6: Figure S2, Table S2). We used a combination of internal and external PCR for validation, similar to the protocol in the 1000 Genomes Project [22]. Out of the 11 loci, six showed clear and distinct bands for SVA insertions. We did not achieve specific amplification for SVA internal products for the remaining loci despite multiple attempts with different PCR conditions (see Method section for detail). This result might partially due to the difficulty in amplifying the complex SVA 5′ region. Although we expect some of these loci are true positives, our current validation results give a minimum true positive rate of 55 % (6/11).

For the six confirmed loci, we then performed individual genotyping to assess the individual genotype calling accuracy (Additional file 5: Figure S2). We consider an individual’s genotype call from our computational pipeline correct if: 1) our pipeline called an SVA insertion and the PCR genotyping validated the insertion (either homozygous or heterozygous); or 2) our pipeline did not call an insertion and the genotyping result is no insertion. In general the individual genotypes are in agreement with computational calls: we achieved 93 % accuracy for individual genotype calls under the relaxed condition for the six loci (Additional file 7: Table S3). For the five loci that are also called under the stringent condition, one locus (Loc 5) has an accuracy of 17 %, primarily due to the under-calling of individuals with the SVA insertion (i.e., false-negative). The remaining four loci have an average accuracy of 96 % (Additional file 6: Table S2).

Next we compared our results with the 1000 Genomes Project phase 3 dataset [22], where 12 samples in our dataset are included. For these 12 overlapping samples, we called 363 SVA insertions and the 1000 Genomes Project called 223 insertions. Based on the primer-binding site position (78–137 in the SVA consensus sequence), 67 SVA insertions in the 1000 Genomes dataset are expected to be amplified by ME-Scan-SVA. Among these 67, 39 loci (58.2 %) were called in our data set. The individual genotype concordance rate for the 39 loci is 78 % (366/468 genotypes). The high genotype concordance rate suggests both datasets have high quality genotype calls for the shared loci.

Because our DNA samples include six parent-offspring trios, we can investigate the inheritance pattern and identify potential *de novo* SVA insertions in the offspring of each trio. To identify *de novo* SVA insertions, SVA insertions in each offspring that are found in parents or shared with unrelated individuals in the dataset (background) were removed. In total, 10 and 3 de novo insertion candidates were identified in the six offspring under the relaxed and stringent cutoffs, respectively. A close inspection showed that all candidate insertion loci are within old retrotransposons or simple repeats in the reference genome. The supporting flanking reads have low mapping quality in general because of the repetitive nature of these regions. Therefore these loci are unlikely to be authentic insertions. Consistent with this observation, two de novo insertion candidates failed validation (Additional file 6: Table S2). Given the SVA retrotransposition rate is estimated to be one in 916 births [39], in six trios the expected chance of identifying a de novo SVA insertion is < 0.01. Therefore, it is not surprising that we did not identify *de novo* SVA insertion in our dataset.

#### Potential functional impact of SVA insertions

**Fig. 6:**
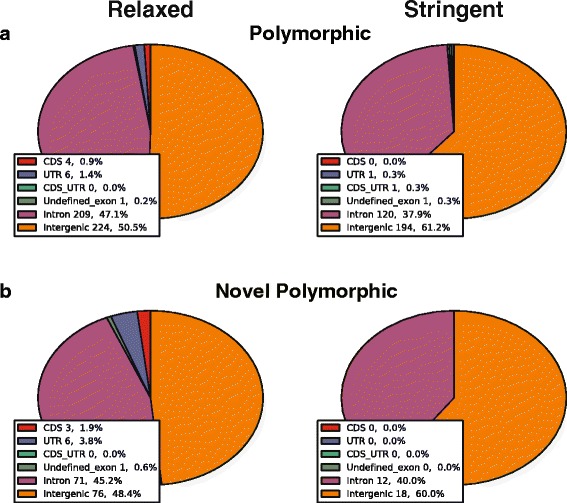
Annotation of SVA insertions. **a** polymorphic SVAs; **b** novel polymorphic SVAs. Results under relaxed and stringent SVA Read cutoffs are shown in the left and right panel, respectively

Next we assessed the potential biological impact of SVA insertions. The insertion loci were intersected with gene annotations from the GENCODE project (Fig. 6). Given less than 5 % of the human genome are annotated as coding sequences (CDS, GENCODE v19), we expect the vast majority of insertions are located in intergenic or intronic regions, assuming a random insertion pattern. As expected, more than 93 % of SVA insertions are located in intergenic or intronic regions and only a small number of insertions overlap exonic regions: polymorphic SVA insertions identified under the relaxed condition intersected with four CDSs, six UTRs (untranslated regions), and one undefined exonic region (Fig. 6a, left). Three of the four CDS insertions were also found in the novel polymorphic dataset, suggesting most exonic insertions identified in this study are novel (Fig. 6b, left). Stringent conditions produced similar results, with only one insertion intersected the CDS region (Fig. 6, right). SVA insertions overlapping CDSs are listed in Additional file 8: Table S5.

**Fig. 7:**
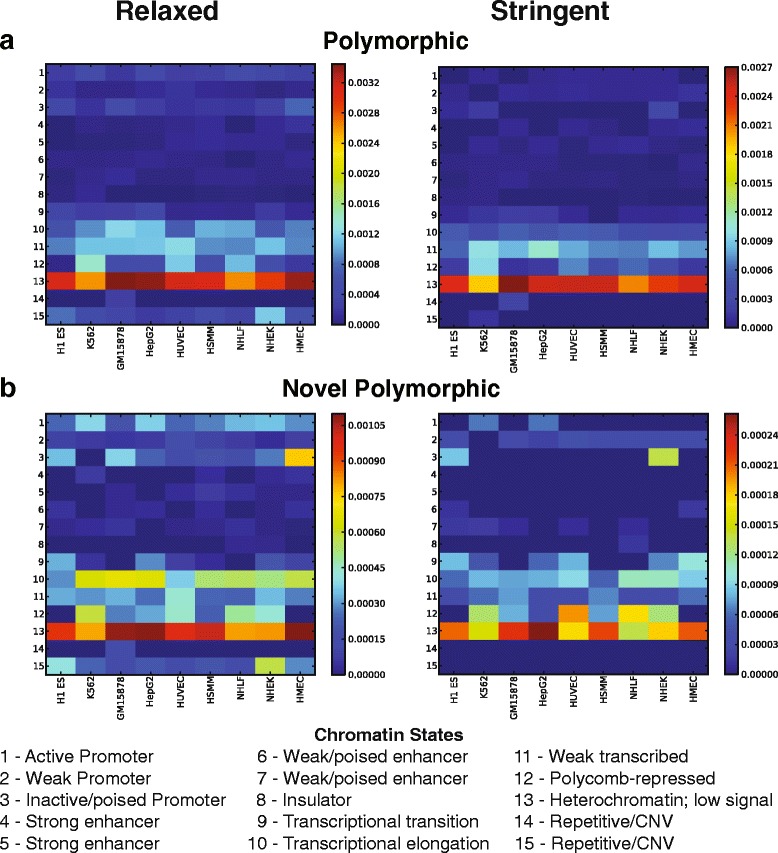
Abundance of SVA insertions in chromatin states. Chromatin state profiles (Y-axis) from nine cell lines (X-axis) were obtained from ChromHMM [44]. For each chromatin state, the normalized number of SVA insertions was shown. **a** polymorphic SVAs; **b** novel polymorphic SVAs. Results under relaxed and stringent SVA Read cutoffs are shown in the left and right panel, respectively

Given most polymorphic SVA insertions are in noncoding regions, we investigated the relationship between SVA insertions and epigenetic modifications. Using the 15 chromatin state profile from nine cell lines as defined by ChromHMM [41], we calculated the normalized number of SVA insertions in each state. The majority of polymorphic SVA insertions are enriched in non- or less- functional genomic regions, especially state 13 (heterochromatin, low signal), suggesting most of these insertions will not affect gene expression (Fig. 7).

## Discussion

As the youngest retrotransposon family in the human genome, SVA insertions are highly polymorphic among human populations and play an important role in gene regulation and contribute to human diseases [19, 23–28]. However, the composite and complex structure of the SVA element has made it difficult to study the insertions using high-throughput sequencing. Here we described ME-Scan-SVA, a protocol for identifying polymorphic SVA insertions in a large number of samples.

Compare to RC-seq [10, 15–17], which uses a probe-based enrichment protocol to selectively enrich for SVAs, ME-Scan-SVA uses a two-round, nested SVA-specific PCR enrichment method. Unlike RC-seq which enriches for both ends of SVA insertions, ME-Scan-SVA only identify the flanking genomic region on the 5′ end of an SVA insertion. This design prevents us from identifying the TSDs of an SVA insertion without follow-up locus-specific sequencing. In addition, because ME-Scan-SVA is designed to preferentially amplify full-length insertions, we will not identify 5′ truncated SVAs that do not have the primer binding sites. Despite of these limitations, this PCR enrichment method has a high specificity: ~94 % of the DNA fragments in the sequencing library passed the SVA Read filtering and are derived from SVA loci. An average 78 % of the total read-pairs passed both SVA Read and Flanking Read filters and we can determine the genomic locations of these potential SVA insertions (Additional file 1: Table S1). This high-specificity for SVA insertions allows us to pool a large number of individuals (e.g., 48) in one sequencing library to save the sequencing cost. Therefore, ME-Scan-SVA is particularly useful in projects that require cost-effective discovery of SVA insertions in a large number of samples.

Another potential future application of the ME-Scan-SVA method is to identify active SVA elements. SVA insertions can carry both 5′ and 3′ flanking sequences during their retrotransposition, in a process known as transduction [18, 26]. The unique genomic sequence carried by the transduction event can be used to trace a new SVA insertion to the active SVA element where the insertion was generated [26]. With the current sequencing length (100 bps), we do not have sufficient flanking sequence to identify most transduction events. In the future, with long read sequencing technology we will be able to identify the transduction events using the ME-Scan-SVA protocol.

## Conclusions

ME-Scan-SVA allows accurate and cost-effective SVA insertions discovery and genotyping. It can be applied in large-scale population studies. It also can be used to study endogenous somatic SVA retrotransposition events in different tissues or developmental stages.

## Methods

### Genomic DNA samples

Genomic DNA samples from 21 individuals were obtained from Coriell Cell Repositories (https://coriell.org/). The samples contain three parent-offspring trios with northern and western European ancestry from the CEPH collection (CEU), three parent-offspring trios from Yoruba in Ibadan, Nigeria (YRI), and three additional YRI individuals. Information including population, family and individual relationships is shown in Table 1.

### Library construction and sequencing

The ME-Scan-SVA libraries were prepared following the ME-Scan protocol described previously [35] with SVA-specific modifications. All the adaptor and primer sequences used in this study were synthesized by Integrated DNA Technologies (Coralville, IA, USA) and are shown in Additional file 9: Table S4.

For each sample, 5 μg genomic DNA was randomly fragmented to about 1 kb in size using Covaris system (Covaris, Woburn, MA, USA) and concentrated using AMPure XP beads (cat. no. A63881, Beckman Coulter, Brea, CA, USA), following the manufacturer’s protocol. The concentrated DNA fragments were then used to construct the sequencing library using KAPA Library Preparation Kits with SPRI solution for Illumina (KAPA Biosystems, Wilmington, MA, USA, cat. no KK8201).

DNA fragments were end-repaired, A-tailed on both ends following the kit protocol. The concentration of the A-tailed DNA was determined using a Nanodrop (Thermo Fisher Scientific, Wilmington, DE, USA). A-tailed DNA fragments were then ligated with adaptors following the protocol of adaptor ligation of KAPA Library Preparation Kit. Each individual was characterized by a unique 6 bp index for downstream identification. The concentration of ligated DNA from each sample was quantified using Nanodrop and the 21 libraries were pooled into one single library with equal concentration. All of the following steps were performed using the pooled library.

SVA-specific first amplification was conducted for 10 cycles with 200 ng of template DNA and 2.5 μl of primer, following the library prep kit amplification protocol (initial denaturation at 98 °C for 45 s, followed by the thermocycling conditions of 98 °C for 15 s, 65 °C for 30 s, and 72 °C for 30 s, and a final extension at 72 °C for 1 min). Size selection was performed on the amplified PCR product using 0.5X of PEG/NaCl SPRI Solution. After size selection, biotinylated SVA-enriched DNA fragments were magnetically separated from other genomic DNA fragments using 5 μl Dynabeads^R^ M-270 Streptavidin (cat. no. 65305, Invitrogen, Life Technologies, Oslo, Norway) following the manufacturer’s protocol. Second amplification was conducted for 12 cycles under the same condition as first amplification, with 24 μl of biotinylated SVA-enriched DNA as template in a 75 μl reaction. The amplified PCR product was electrophoresed at 120 volts for 90 min on a 2 % NuSieve^R^ GTG^R^ Agarose gel (cat. no. 50080, Lonza, Rockland, Maine, USA). Fragments around 500 bp were size selected and purified using Wizard SV Gel and PCR Clean-up system (cat. no. A9281, Promega, Madison, WI, USA).

Before the library was sequenced, its fragment size and concentration was determined using Bioanalyzer and quantitative PCR by the RUCDR Infinite Biologics (Piscataway, NJ, USA). The library was sequenced using the Illumina Hiseq 2000 with 100PE format at RUCDR Infinite Biologics.

### Computational analysis

The computational analysis pipeline was constructed using a combination of bash and python codes. The codes are available at https://github.com/JXing-Lab/ME-SCAN-SVA/.

Briefly, ncbi-blast-2.2.28+ [37] was used to compare SVA sequence in the SVA Read to the SVA consensus sequence to generate BLAST bit-scores. BWA-MEM (ver. 0.7.5a) [38] was used to map Flanking Read against the human reference genome (hg19). Samtools-1.1 [42] were used to count the number of Flanking Read mapped to the human reference genome in each individual for TPM calculation. BEDTools (Ver. 2.16.2) [43] was used to cluster all mapped reads in a region and generate a list of candidate insertion loci for downstream analyses. Using customized python and bash codes, results from all applications were integrated into the current pipeline.

Known polymorphic loci were obtained from the Database of Retrotransposon Insertion Polymorphisms (dbRIP, [40]), HuRef genome [39], and the 1000 Genomes data [5, 22]. Gene annotation was obtained from GENCODE (Release v19). Chromatin state profiles from nine cell lines were obtained from ChromHMM [44]. For each chromatin state, the normalized number of SVA insertions (number of insertions divided by total number of locations in each state) was calculated.

### Genotyping PCR for validation

Three separate PCR reactions were performed for each of the 13 loci (11 polymorphic and 2 de novo candidates): one outside primer with two different internal primers (SVA_1 internal, and SVA_2 internal, Additional file 9: Table S4) in two reactions and external primer pair in one reaction (Additional file 5: Figure S2B). Because the 5′ end of an SVA element contains a (CTCCCT)_n_ simple repeat region and an *Alu* region that shares homology with *Alu* elements, non-specific amplifications occurred at many loci. In these cases different DNA polymerases, annealing temperatures, PCR buffers (standard and high GC buffer), PCR additive betaine, and primer locations were attempted. However, for 7 loci (5 polymorphic, 2 de novo) no specific internal/external amplification was achieved. The PCRs were performed using One Taq hot start DNA polymerase with GC buffer (cat. no. M0481, New England Biolabs, Ipswich, MA, USA). The thermocycling condition is: an initial denaturation at 94 °C for 30 s, followed by 30 cycles of 94 °C for 30 s, a locus-specific annealing temperature (Additional file 6: Table S2) for 1 min, and 68 °C for 3 min, followed by a final extension at 68 °C for 3 min The PCR products were electrophoresed at 300 volts for 25 min on a 1.5 % GenePure LE Agarose gel (cat. no. E-3120-500, BioExpress, Kaysville, UT, USA). For loci that showed clear and distinct bands, individual genotyping was performed. The DNA fragments of all these loci from at least one individual were validated by Sanger sequencing.

## Abbreviations

CDS, coding sequence; MEIs, Mobile element insertions; ME-Scan, mobile element scanning; pMEIs, polymorphic mobile element insertions; TPM, tags per million; UR, unique reads; UTR, untranslated region; VNTR, variable number of tandem repeats

## Additional files

Additional file 1: Table S1. Number of passed filter reads in each sample. (XLSX 16 kb) Additional file 2: Figure S1. Sensitivity Analysis. The sensitivity for identifying fixed SVA insertions under different TPM and UR cutoffs in each individual. The sensitivity is shown as the percentage of fixed insertions identified. (A) Relaxed SVA Read cutoff; (B) stringent SVA Read cutoff. (PDF 2419 kb) Additional file 3: Polymorphic SVA candidate loci with relaxed SVA Read cutoff. (TXT 89 kb) Additional file 4: Polymorphic SVA candidate loci with stringent SVA Read cutoff. (TXT 60 kb) Additional file 5: Figure S2. Individual genotypes of polymorphic SVA insertions. For each individual, three PCR reactions were performed: SVA_1 + outside primer; SVA_2 + outside primer; and outside primer pairs. (A) Genotyping results of Locus 3. Each individual ID is labelled on the top of the lane. For a sample with a homozygous no insertion genotype (e.g., NA12873), the two internal-external primer pairs (SVA_1 + 3R; SVA_2 + 3R) are expected to have no PCR product, and the outside primer pairs (3F + 3R) is expected to amplify the genomic region without SVA insertion. The expected empty (i.e., no insertion) product size for the outside primer pairs is 566 bps. For a sample with a heterozygous insertion genotype (e.g., NA12872), all three reactions will have PCR products. The expected PCR product sizes for the internal-external primer pairs are uncertain because of the unknown size of the SVA 5′ (CCCTCT)_n_ hexamer simple repeat region. For a sample with a homozygous insertion genotype (e.g., NA18504), the two internal-external primer pairs are expected to have PCR products, and the outside primer pairs is expected to either have no amplification or a large PCR product (SVA + flanking sequence). (B) PCR primer location diagram for Locus 3. The primers are represented by arrows. The color scheme is same as Additional file 2: Figure S1. (C-G) Individual genotyping results of Locus 1, 4, 5, 6, and 9. The expected empty product sizes are shown in Additional file 6: Table S2. (PDF 5287 kb) Additional file 6: Table S2. Candidate SVA insertion loci subjected to PCR validation. (XLSX 12 kb) Additional file 7: Table S3. Individual genotypes of validated loci. (XLSX 16 kb) Additional file 8: Table S5. SVA insertions overlapping protein coding regions. (XLSX 11 kb) Additional file 9: Table S4. Oligo and primers used in this study. (XLSX 10 kb)

## References

[1] de Koning AP, Gu W, Castoe TA, Batzer MA, Pollock DD (2011). Repetitive elements may comprise over two-thirds of the human genome. PLoS Genet.

[2] Cordaux R, Batzer MA (2009). The impact of retrotransposons on human genome evolution. Nat Rev Genet.

[3] Beck CR, Garcia-Perez JL, Badge RM, Moran JV (2011). LINE-1 elements in structural variation and disease. Annu Rev Genomics Hum Genet.

[4] Hancks DC, Kazazian HH (2012). Active human retrotransposons: variation and disease. Curr Opin Genet Dev.

[5] Stewart C, Kural D, Stromberg MP, Walker JA, Konkel MK, Stutz AM, Urban AE, Grubert F, Lam HY, Lee WP (2011). A comprehensive map of mobile element insertion polymorphisms in humans. PLoS Genet.

[6] Nishihara H, Okada N (2008). Retroposons: genetic footprints on the evolutionary paths of life. Methods Mol Biol.

[7] Ray DA, Xing J, Salem AH, Batzer MA (2006). SINEs of a nearly perfect character. Syst Biol.

[8] Xing J, Witherspoon DJ, Ray DA, Batzer MA, Jorde LB (2007). Mobile DNA elements in primate and human evolution. Am J Phys Anthropol.

[9] Xing J, Witherspoon DJ, Jorde LB (2013). Mobile element biology: new possibilities with high-throughput sequencing. Trends Genet.

[10] Baillie JK, Barnett MW, Upton KR, Gerhardt DJ, Richmond TA, De Sapio F, Brennan PM, Rizzu P, Smith S, Fell M (2011). Somatic retrotransposition alters the genetic landscape of the human brain. Nature.

[11] Witherspoon DJ, Xing J, Zhang Y, Watkins WS, Batzer MA, Jorde LB (2010). Mobile element scanning (ME-Scan) by targeted high-throughput sequencing. BMC Genomics.

[12] Iskow RC, McCabe MT, Mills RE, Torene S, Pittard WS, Neuwald AF, Van Meir EG, Vertino PM, Devine SE (2010). Natural mutagenesis of human genomes by endogenous retrotransposons. Cell.

[13] Ewing AD, Kazazian HH (2010). High-throughput sequencing reveals extensive variation in human-specific L1 content in individual human genomes. Genome Res.

[14] Witherspoon DJ, Zhang Y, Xing J, Watkins WS, Ha H, Batzer MA, Jorde LB (2013). Mobile element scanning (ME-Scan) identifies thousands of novel Alu insertions in diverse human populations. Genome Res.

[15] Sanchez-Luque FJ, Richardson SR, Faulkner GJ (2016). Retrotransposon Capture Sequencing (RC-Seq): A Targeted, High-Throughput Approach to Resolve Somatic L1 Retrotransposition in Humans. Methods Mol Biol.

[16] Klawitter S, Fuchs NV, Upton KR, Munoz-Lopez M, Shukla R, Wang J, Garcia-Canadas M, Lopez-Ruiz C, Gerhardt DJ, Sebe A (2016). Reprogramming triggers endogenous L1 and Alu retrotransposition in human induced pluripotent stem cells. Nat Commun.

[17] Shukla R, Upton KR, Munoz-Lopez M, Gerhardt DJ, Fisher ME, Nguyen T, Brennan PM, Baillie JK, Collino A, Ghisletti S (2013). Endogenous retrotransposition activates oncogenic pathways in hepatocellular carcinoma. Cell.

[18] Wang H, Xing J, Grover D, Hedges DJ, Han K, Walker JA, Batzer MA (2005). SVA elements: a hominid-specific retroposon family. J Mol Biol.

[19] Ostertag EM, Goodier JL, Zhang Y, Kazazian HH (2003). SVA elements are nonautonomous retrotransposons that cause disease in humans. Am J Hum Genet.

[20] Raiz J, Damert A, Chira S, Held U, Klawitter S, Hamdorf M, Lower J, Stratling WH, Lower R, Schumann GG (2012). The non-autonomous retrotransposon SVA is trans-mobilized by the human LINE-1 protein machinery. Nucleic Acids Res.

[21] Hancks DC, Goodier JL, Mandal PK, Cheung LE, Kazazian HH (2011). Retrotransposition of marked SVA elements by human L1s in cultured cells. Hum Mol Genet.

[22] Sudmant PH, Rausch T, Gardner EJ, Handsaker RE, Abyzov A, Huddleston J, Zhang Y, Ye K, Jun G, Hsi-Yang Fritz M (2015). An integrated map of structural variation in 2,504 human genomes. Nature.

[23] Kwon YJ, Choi Y, Eo J, Noh YN, Gim JA, Jung YD, Lee JR, Kim HS (2013). Structure and Expression Analyses of SVA Elements in Relation to Functional Genes. Genome Inform.

[24] Xing J, Wang H, Belancio VP, Cordaux R, Deininger PL, Batzer MA (2006). Emergence of primate genes by retrotransposon-mediated sequence transduction. Proc Natl Acad Sci U S A.

[25] Hancks DC, Kazazian HH (2010). SVA retrotransposons: Evolution and genetic instability. Semin Cancer Biol.

[26] Damert A, Raiz J, Horn AV, Lower J, Wang H, Xing J, Batzer MA, Lower R, Schumann GG (2009). 5′-Transducing SVA retrotransposon groups spread efficiently throughout the human genome. Genome Res.

[27] Hancks DC, Ewing AD, Chen JE, Tokunaga K, Kazazian HH (2009). Exon-trapping mediated by the human retrotransposon SVA. Genome Res.

[28] Quinn JP, Bubb VJ. SVA retrotransposons as modulators of gene expression. Mobile Genet Elem. e32102;4. 10.4161/mge.32102 PMC4114917 25077041

[29] van der Klift HM, Tops CM, Hes FJ, Devilee P, Wijnen JT (2012). Insertion of an SVA element, a nonautonomous retrotransposon, in PMS2 intron 7 as a novel cause of Lynch syndrome. Hum Mutat.

[30] Conley ME, Partain JD, Norland SM, Shurtleff SA, Kazazian HH (2005). Two independent retrotransposon insertions at the same site within the coding region of BTK. Hum Mutat.

[31] Wilund KR, Yi M, Campagna F, Arca M, Zuliani G, Fellin R, Ho YK, Garcia JV, Hobbs HH, Cohen JC (2002). Molecular mechanisms of autosomal recessive hypercholesterolemia. Hum Mol Genet.

[32] Nakamura Y, Murata M, Takagi Y, Kozuka T, Nakata Y, Hasebe R, Takagi A, Kitazawa J, Shima M, Kojima T (2015). SVA retrotransposition in exon 6 of the coagulation factor IX gene causing severe hemophilia B. Int J Hematol.

[33] Vogt J, Bengesser K, Claes KB, Wimmer K, Mautner VF, van Minkelen R, Legius E, Brems H, Upadhyaya M, Hogel J (2014). SVA retrotransposon insertion-associated deletion represents a novel mutational mechanism underlying large genomic copy number changes with non-recurrent breakpoints. Genome Biol.

[34] Platt RN, Zhang Y, Witherspoon DJ, Xing J, Suh A, Keith MS, Jorde LB, Stevens RD, Ray DA (2015). Targeted Capture of Phylogenetically Informative Ves SINE Insertions in Genus Myotis. Genome Biol Evol.

[35] Ha H, Wang N, Xing J. Library construction for high-throughput mobile element identification and genotyping. Methods Mol Biol. 2015. [Epub ahead of print]. 10.1007/7651_2015_265 26025622

[36] Jurka J, Kapitonov VV, Pavlicek A, Klonowski P, Kohany O, Walichiewicz J (2005). Repbase Update, a database of eukaryotic repetitive elements. Cytogenet Genome Res.

[37] Altschul SF, Gish W, Miller W, Myers EW, Lipman DJ (1990). Basic local alignment search tool. J Mol Biol.

[38] Li H, Durbin R (2009). Fast and accurate short read alignment with Burrows-Wheeler transform. Bioinformatics.

[39] Xing J, Zhang Y, Han K, Salem AH, Sen SK, Huff CD, Zhou Q, Kirkness EF, Levy S, Batzer MA (2009). Mobile elements create structural variation: analysis of a complete human genome. Genome Res.

[40] Wang J, Song L, Grover D, Azrak S, Batzer MA, Liang P (2006). dbRIP: a highly integrated database of retrotransposon insertion polymorphisms in humans. Hum Mutat.

[41] Ernst J, Kheradpour P, Mikkelsen TS, Shoresh N, Ward LD, Epstein CB, Zhang X, Wang L, Issner R, Coyne M (2011). Mapping and analysis of chromatin state dynamics in nine human cell types. Nature.

[42] Li H, Handsaker B, Wysoker A, Fennell T, Ruan J, Homer N, Marth G, Abecasis G, Durbin R, Genome Project Data Processing S (2009). The Sequence Alignment/Map format and SAMtools. Bioinformatics.

[43] Quinlan AR, Hall IM (2010). BEDTools: a flexible suite of utilities for comparing genomic features. Bioinformatics.

[44] Ernst J, Kellis M (2012). ChromHMM: automating chromatin-state discovery and characterization. Nat Methods.

