# Supplementary figures and images for: Identification of polymorphic SVA retrotransposons using a mobile element scanning method for SVA (ME-Scan-SVA)
Source: Mob DNA. 2016 Jul 30;7:15. doi: 10.1186/s13100-016-0072-x (PMC4967303; doi:10.1186/s13100-016-0072-x)

**A****Locus 3 (chr3:112466690)**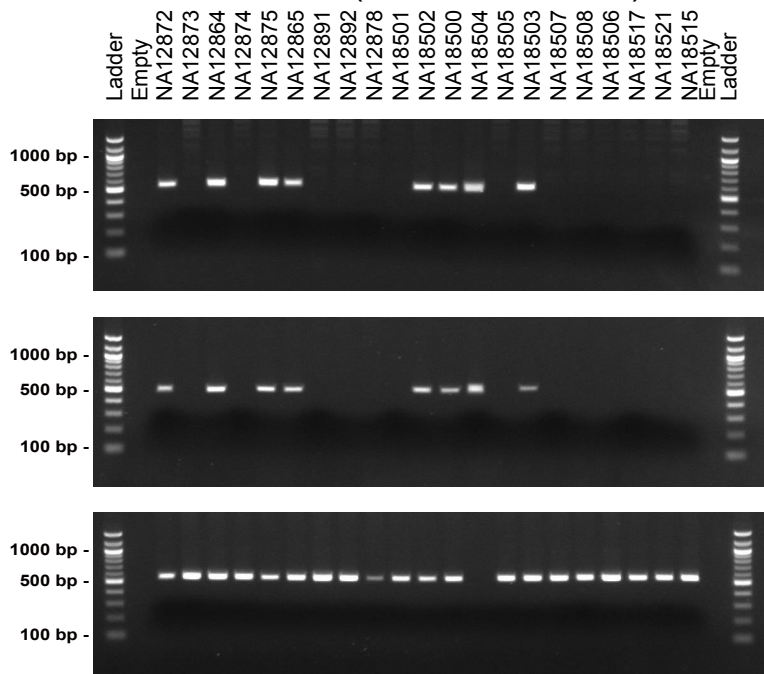**B**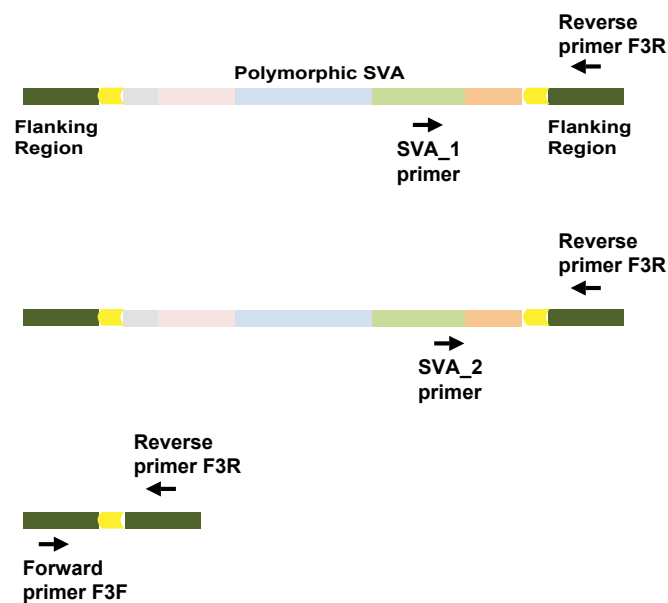**C****Locus 1 (chr1:112182028)**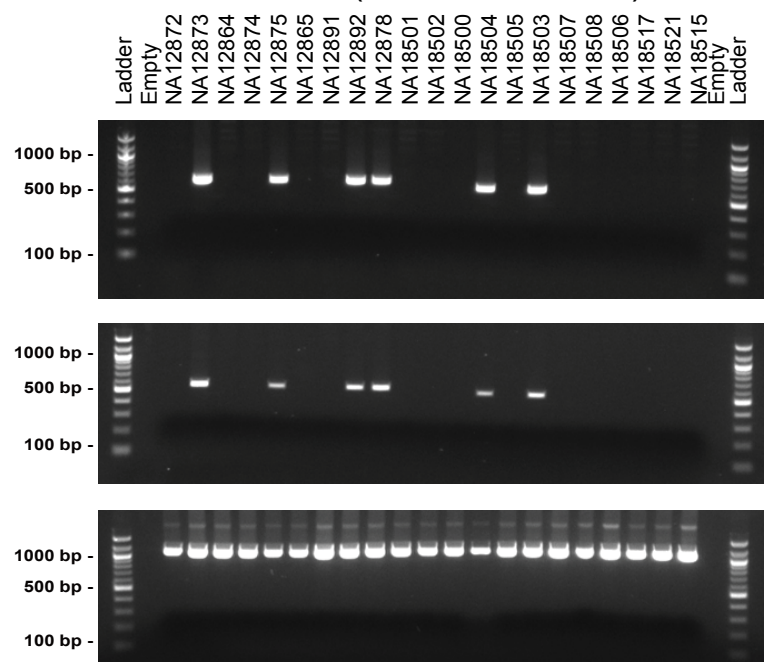**D****Locus 4 (chr3:183433065)**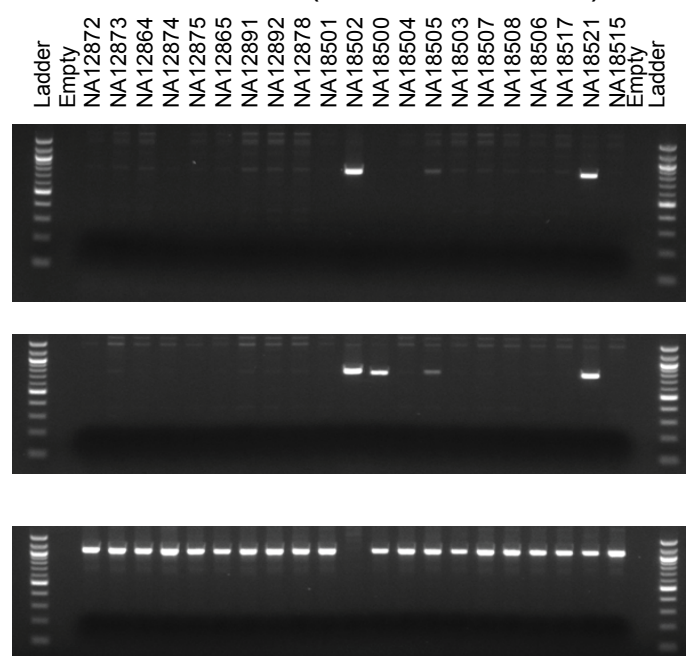

## E Locus 5 (chr4:190684483)

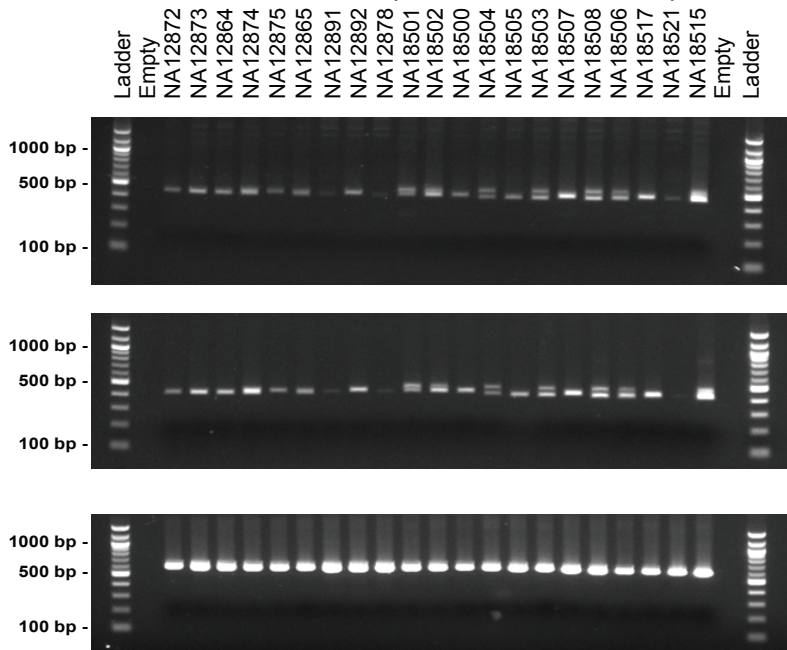

## F Locus 6 (chr5:39511976)

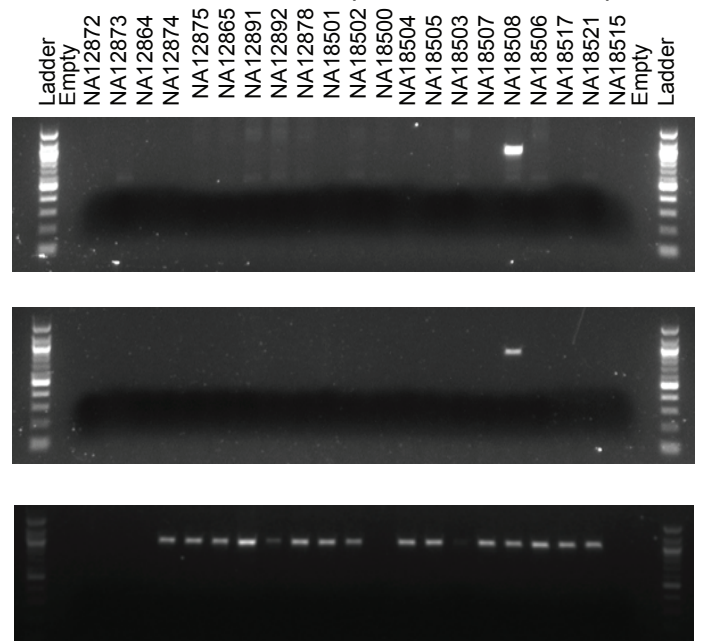

## G Locus 9 (chr11:33828713)

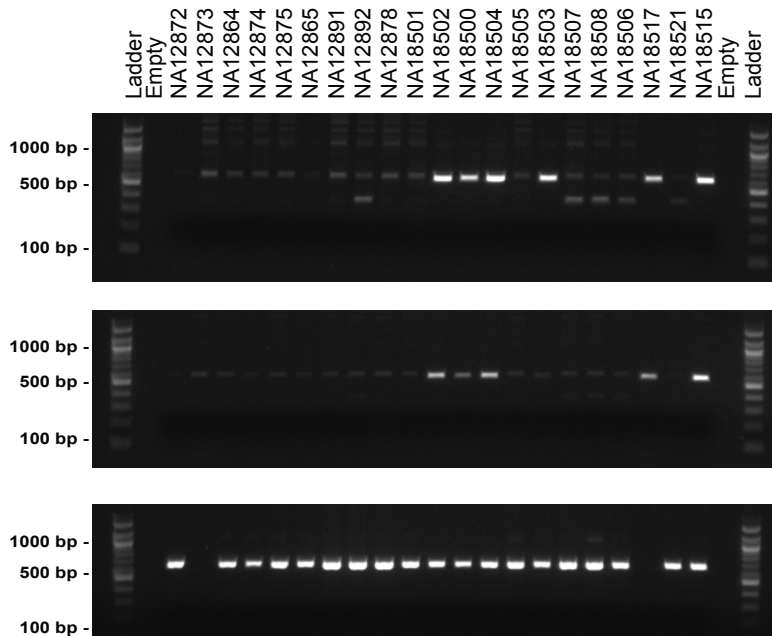

Supplement: Additional file 5: Figure S2. — Individual genotypes of polymorphic SVA insertions. For each individual, three PCR reactions were performed: SVA_1 + outside primer; SVA_2 + outside primer; and outside primer pairs. (A) Genotyping results of Locus 3. Each individual ID is labelled on the top of the lane. For a sample with a homozygous no insertion genotype (e.g., NA12873), the two internal-external primer pairs (SVA_1 + 3R; SVA_2 + 3R) are expected to have no PCR product, and the outside primer pairs (3F + 3R) is expected to amplify the genomic region without SVA insertion. The expected empty (i.e., no insertion) product size for the outside primer pairs is 566 bps. For a sample with a heterozygous insertion genotype (e.g., NA12872), all three reactions will have PCR products. The expected PCR product sizes for the internal-external primer pairs are uncertain because of the unknown size of the SVA 5′ (CCCTCT)n hexamer simple repeat region. For a sample with a homozygous insertion genotype (e.g., NA18504), the two internal-external primer pairs are expected to have PCR products, and the outside primer pairs is expected to either have no amplification or a large PCR product (SVA + flanking sequence). (B) PCR primer location diagram for Locus 3. The primers are represented by arrows. The color scheme is same as Additional file 2: Figure S1. (C-G) Individual genotyping results of Locus 1, 4, 5, 6, and 9. The expected empty product sizes are shown in Additional file 6: Table S2. (PDF 5287 kb) [file 13100_2016_72_MOESM5_ESM.pdf]
