# Supplementary material for: Identification of polymorphic SVA retrotransposons using a mobile element scanning method for SVA (ME-Scan-SVA)
Source: Mob DNA. 2016 Jul 30;7:15. doi: 10.1186/s13100-016-0072-x (PMC4967303; doi:10.1186/s13100-016-0072-x)
Supplement: Additional file 2: Figure S1. — Sensitivity Analysis. The sensitivity for identifying fixed SVA insertions under different TPM and UR cutoffs in each individual. The sensitivity is shown as the percentage of fixed insertions identified. (A) Relaxed SVA Read cutoff; (B) stringent SVA Read cutoff. (PDF 2419 kb) [file 13100_2016_72_MOESM2_ESM.pdf]

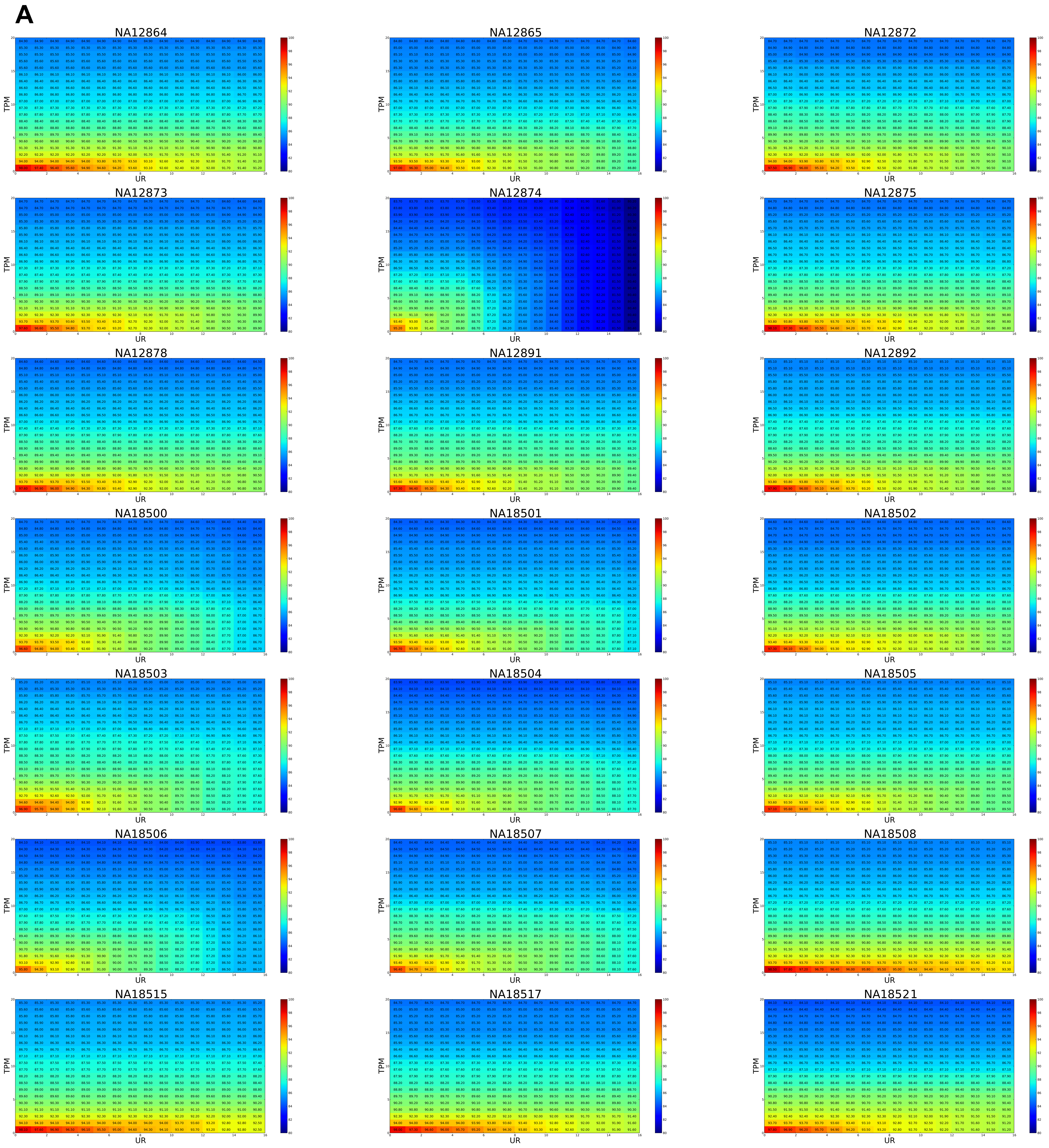
A
NA12864
NA12865
NA12872
NA12873
NA12874
NA12875
NA12878
NA12891
NA12892
NA18500
NA18501
NA18502
NA18503
NA18504
NA18505
NA18506
NA18507
NA18508
NA18515
NA18517
NA18521
TPM
UR

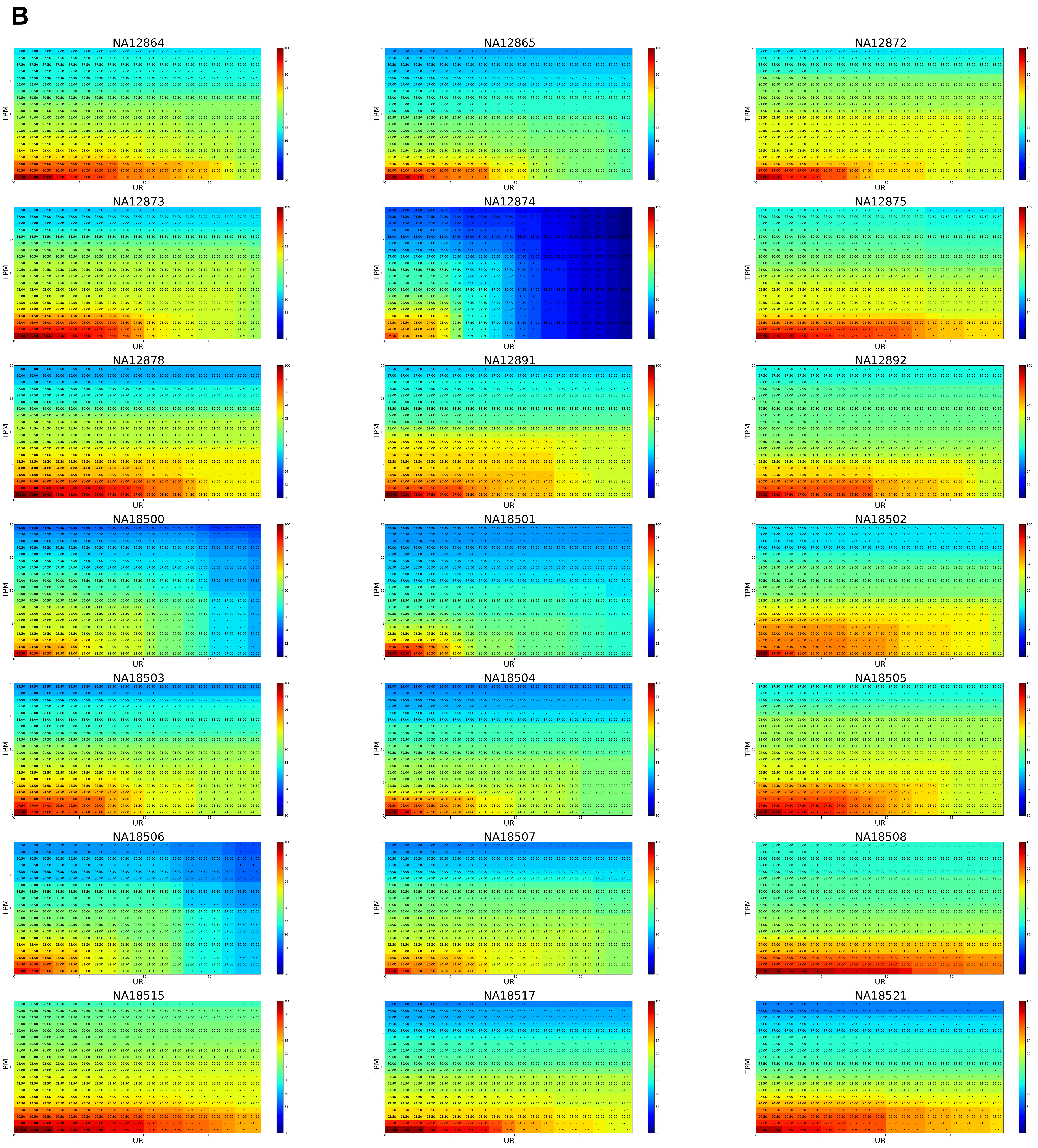
B
NA12864
NA12865
NA12872
NA12873
NA12874
NA12875
NA12878
NA12891
NA12892
NA18500
NA18501
NA18502
NA18503
NA18504
NA18505
NA18506
NA18507
NA18508
NA18515
NA18517
NA18521
TPM
UR
